# A Microfluidic pH Measurement Device with a Flowing Liquid Junction

**DOI:** 10.3390/s17071563

**Published:** 2017-07-04

**Authors:** Akira Yamada, Miho Suzuki

**Affiliations:** Department of Mechanical Engineering, Graduate School of Engineering, Aichi Institute of Technology, Toyota 470-0392, Japan; p17710pp@aitech.ac.jp

**Keywords:** pH, ISFET, pH-FET, microfluidic device, liquid junction, flowing junction

## Abstract

The pH values of aqueous solutions are conventionally measured with pH-sensitive electrodes such as glass electrodes or ion-sensitive field-effect transistors (ISFETs) used in conjunction with Ag/AgCl reference electrodes and KCl solutions. The speed of pH measurement with these systems can be deficient, however, as the glass electrode responds slowly during measurements of sample solutions with low buffering capacities. Our group has constructed a new pH measurement system using a microfluidic device and ISFET sensors. The device has a channel with two inlets and one outlet, with a junction connected to a Y-shaped channel on the same plane. Two ISFET sensors and an Ag/AgCl pseudo reference electrode are fitted into the channel to construct a differential measurement device. A sample solution and baseline solution supplied into the inlets by gravity-driven pumps form a flowing liquid junction during measurement. The small size and fast response of the ISFET sensors enable measurement of about 2.0 mL of sample solution over a measurement period of 120 s. The 90% response time is within 2 s. The calibrated sensor signal exhibits a wide range (pH 1.68–10.0) of linearity with a correlation factor of 0.9997. The measurement error for all solutions tested, including diluted solutions, was 0.0343 ± 0.0974 pH (average error ± standard deviation (S.D.), n = 42). The new device developed in this research will serve as an innovative technology in the field of potentiometry.

## 1. Introduction

The pH scale is a numeric index used to specify the acidity and alkalinity of aqueous solutions. One of the domains in which it is frequently applied is environmental science. Precise pH values can be obtained by taking potentiometric measurements with devices such as glass electrodes [1] or ion-sensitive field-effect transistors (ISFETs) [2,3] used in conjunction with reference electrodes [4]. The most commonly applied pH electrode is a glass type used together with an Ag/AgCl reference electrode and KCl solution [4]. Though traditional and reliable, the glass electrode method still has shortcomings. The long response time of the glass electrode precludes a shortened measurement time and leads to large measurement error and low reproducibility [4,5,6]. These shortcomings become especially problematic in the measurement of sample solutions with low buffering capacities [4,5,6]. Environmental waters, such as natural water and water released from industrial plants, often have low buffering capacities. Precise measurements of their pH values using glass electrodes may therefore require long periods to complete. 

The ISFET sensors developed with semiconductor technology have many advantages over glass electrodes [2,3]. Glass electrodes and ISFETs differ in both size and responsiveness. The former are difficult to downsize without sacrificing the electrical characteristics. ISFET sensors, in turn, have smaller electrodes and perform with superior responsiveness.

The liquid junction (LJ) is essential in potentiometric measurements. The KCl solution used in the reference electrode keeps the electromotive force of the Ag/AgCl electrode constant [4,7]. LJ devices based on several principles have been developed, including a ceramic type, double junction type, free diffusion junction type, and flowing junction type [4,6,8,9,10]. The first three types mentioned can be used in combination with electrodes [6,8,11]. While the fourth type, the flowing LJ, obtains stable measurement conditions due to the flow of the solution, the outer body is too large to fit into a practical measurement apparatus [4]. 

Our group has developed a flow-through-type differential sensor probe consisting of two ISFET sensors [12,13]. The sensor probe is integrated into an automated pH measurement system (Auto-pH) consisting of XYZ stages, a pump, electric circuit, and PC [14]. In trial measurements, the system performed highly accurate, high-speed measurements of 96 small-volume samples within a short time [14]. Special adjustments were necessary in some cases, however, as solutions with low buffering capacities required spatiotemporal control of the LJ to manage the microfluidic behavior in order to shorten the response time and improve the measurement accuracy [15].

Microfluidic technologies are used in applications in chemistry, biochemistry, molecular biology, and medicine [16,17]. A micro channel of at least one dimension smaller than a few mm has an extremely low Reynolds number (Re), which results in a laminar fluid flow without turbulence. If two flowing fluids meet in a Y-shaped channel with two inlets, an unmixed sharp boundary formed between the fluids acts as an LJ, especially under a flowing condition.

Microfluidic devices for pH measurement have been reported in several papers. On the environmental front, a self-calibrating device fitted with an ISFET has been used to measure pH in situ at ocean depths [18]. In a study from Europe, a colorimetric microfluidic pH sensor was developed for autonomous seawater measurement [19]. In the field of biology, local pH values were measured in a microfluidic device using a resin gel-microbead material with a pH indicator [20].

In the present investigation we constructed and evaluated a new pH measurement system using a microfluidic device and ISFET sensors. A flowing LJ within the microfluidic device enabled stable measurements. The present device exhibited a rapid response in pH measurements of sample solutions with low buffering capacities. These features enabled superior measurement accuracy within a pH range of 1.68–10.0. The present pH measurement system can be applied in the environmental sciences. 

## 2. Materials and Methods 

### 2.1. Measurement Principle 

**Figure 1:**
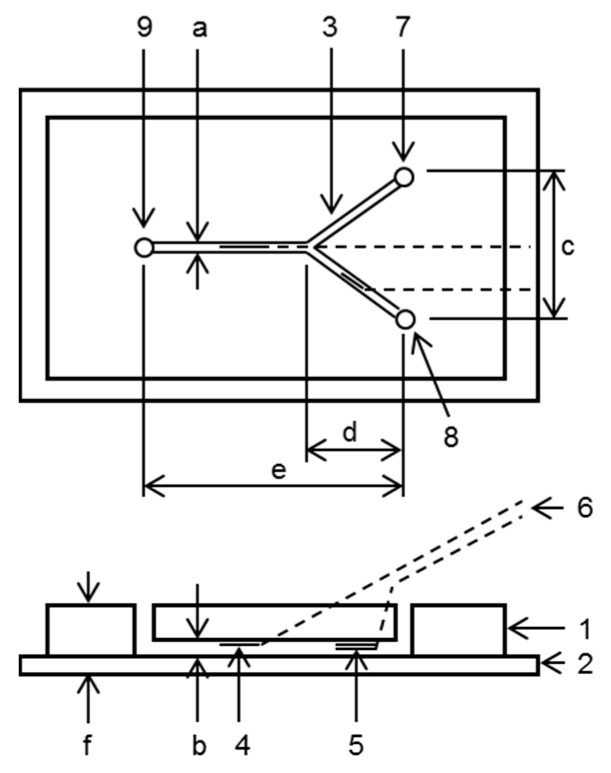
A schematic representation of the microfluidic pH measurement device made of polydimethylsiloxane (PDMS) and a glass slide. 1: pH measurement device body; 2: Glass slide; 3: Channel; 4: Measuring pH-FET (pH-FET_m_); 5: Reference pH-FET (pH-FET_r_) and pseudo-reference electrode; 6: Lead wires; 7: Sample solution inlet; 8: Baseline solution (BLS) inlet; 9: Outlet. Sizes (mm): a (1.0); b (0.5); c (17); d (12); e (32); f (6–8).

Figure 1 shows a schematic representation of the microfluidic device for pH measurement. The device consists of a channel with two-inlets and one outlet. Two pH-sensitive field-effect transistor (pH-FET) sensors and an Ag/AgCl electrode are installed into the channel. The pH-FET sensor for measurement (pH-FET_m_) is set downstream of the junction, and the pH-FET for reference (pH-FET_r_) and Ag/AgCl electrode are set near the inlet for the baseline solution (BLS). The measurements are performed under a flowing condition.

**Figure 2:**
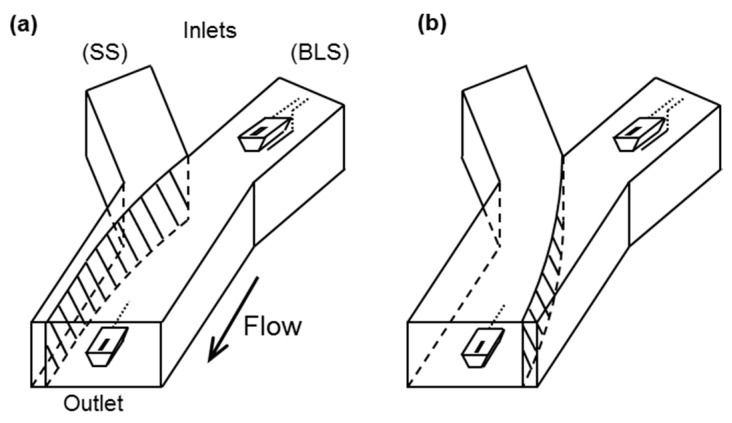
Measurement principle using a Y-shaped channel and pH-FET sensors. The flowing paths of the two solutions at (**a**) the initialization stage before measurement and (**b**) during measurement. The hatched region shows the area of contact between the two solutions, a region that serves as a liquid junction during measurement.

**Figure 3:**
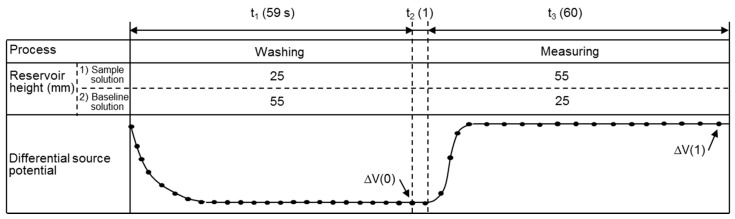
Time course of one sample-measurement cycle: t_1_, washing the pH-FET_m_ sensor and channel inside; t_2_, switching the reservoir heights; t_3_, measurement.

Figure 2 indicates the measurement principle using a differential formula. The present system obtains the source potential for the measurement (V_m_) and reference (V_r_) pH-FETs with respect to the Ag/AgCl electrode under the two flow path conditions shown in Figure 2a,b. Figure 3 shows the time course of one measurement cycle. First, the two pH-FET sensors are immersed in the BLS and a differential sensor signal (ΔV(0) = V_m_ – V_r_) is recorded in the final second of the washing period (Figure 2a). Next, the flow paths of the two solutions are switched to immerse the pH-FET_m_ in the sample solution, and a differential sensor signal (ΔV(1) = V_m_ – V_r_) is recorded in the final second of the measurement period (Figure 2b). The pH-FET_m_ and Ag/AgCl electrode are electrically connected through an LJ of the two solutions. A double differential source potential is defined as ΔΔV = ΔV(1) – ΔV(0), and the pH values correspond to the ΔΔV values. A differential measurement system is thus constructed.

The flow velocity (v (cm/s)) and Re were calculated as follows: v = Q/wh and Re = wv/n, where w and h are the width and height (cm) of the flow path, Q is the volume flow (ml/s), and *n* is the kinetic viscosity (cm^2^/s) at 25 °C. The v of the flowing fluid and Re during measurement were estimated to be 0.067 cm/s and 0.75, respectively. The source potential of the two pH-FETs, V_m_ and V_r_, were not affected by the flow rate under the measurement condition. While the total flow rates of the sample solution and BLS remained almost constant during measurement, the flow velocity around the pH-FET_r_ sensor and Ag/AgCl electrode differed between the washing and measurement periods (Figure 2 and Figure 3).

### 2.2. Fabrication of the Microfluidic pH Measurement Device

**Figure 4:**
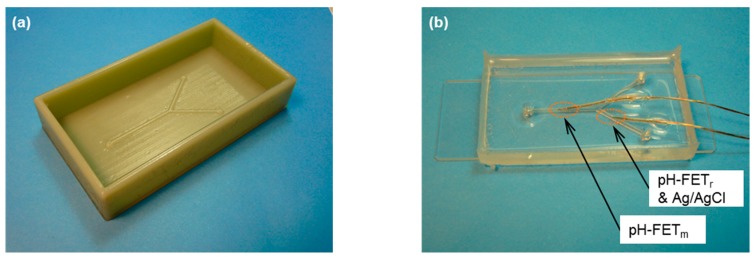
(**a**) A mold fabricated using a rapid prototyping system. External form: 36 × 63 × 12 mm. (**b**) Overview of a microfluidic device with pH-FET sensors. External form of the main body: 32 × 76 × 7 mm.

The microfluidic device for pH measurement was composed of a concave Y-shaped channel made from polydimethylsiloxane (PDMS) and a glass slide. The device body was constructed using soft lithography [16]. A convex mold with a bank was designed by three-dimensional computer-aided design (3D-CAD) and fabricated using a rapid prototyping system (Objet 500, Connex, Stratasys Ltd., Rehovot, Israel) (Figure 4a). A transparent liquid PDMS was poured into the mold, hardened overnight, and removed from the mold. As shown in Figure 4b, the channel side of the PDMS structure and glass slide were hydrophilized by plasma irradiation (PDC-32G, Harric Plasma, NY, USA) and adhered together. The holes for the inlets and outlet of the device body were punched by a hole puncher (ϕ = 3 mm). The pH-FET sensors [21,22] and Ag/AgCl electrode were inserted into the channel through the tiny holes. The gap between each hole and lead wire was filled with a silicone adhesive to prevent the solution from leaking. Each pH-FET sensor measured 5.5 × 0.45 × 0.2 mm.

### 2.3. Measurement System 

**Figure 5:**
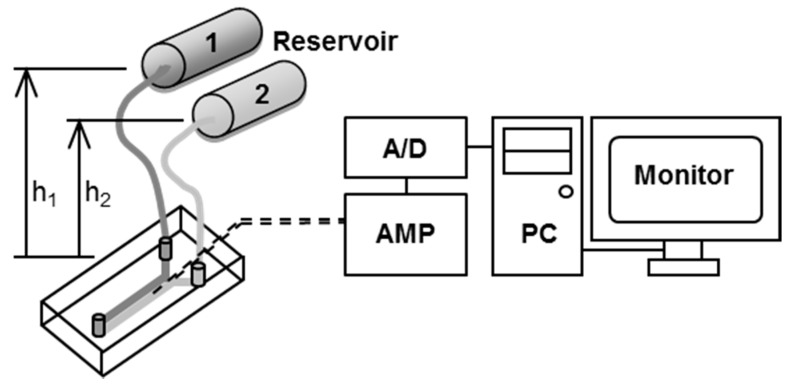
Schematics of the measurement system. Reservoir 1, sample solution; Reservoir 2, BLS. The h_1_ and h_2_ are the heights of the reservoirs.

Figure 5 shows schematics of the measurement system used in this work. The system was composed of a microfluidic device, two reservoirs, an electric circuit for pH-FET sensors, a 16-bit analog-to-digital converter (CONTEC, Osaka, Japan), and a PC (DOS/V). In our trial measurements, the source potentials of the pH-FETs were taken at 1.0 s intervals. The reservoir was connected to the inlet of the microfluidic device by a tube. The solutions were fed from the reservoirs to the inlets by hydrostatic pressure caused by gravity, and the flow rates were adjusted by changing the heights of the reservoirs. Just before measurement, the reservoirs were set above the measurement device at heights of h_1_ = 25 and h_2_ = 55 mm and filled with sample solution and BLS, respectively (Figure 3 and Figure 5). Each reservoir consisted of a narrow (ϕ = 4 mm) pipe kept parallel to the floor to keep the hydrostatic pressure constant during measurement. We derived values for the control parameters to shorten the time required for measurement and improve the measurement accuracy. The present device consumed 2.0 mL of sample solution per measurement and completed the cycle in 120 s: 59 s for washing, 1 s for switching the solutions, and 60 s for measurement (Figure 3).

### 2.4. Measurements, Calibration, and Evaluation of Linearity 

The procedures for calibration and measurement were the same (Figure 3). To start the cycle, 2.0 mL of sample solution and 2.0 mL of BLS were poured into the respective reservoirs. Pinch cocks were then opened to commence the solution flows. ΔΔV was read out three times for each pH standard solution and each sample solution.

Six standard solutions (described later) were measured for calibration and the pH values were derived from the following equation. 

pH = pH_0_ + ΔΔV/k, (1)

The pH_0_ and k values were determined by linear approximation and the linearity of the calibration function was evaluated by a correlation factor.

### 2.5. Evaluation of Response Times and Measurement Accuracy 

The response times and measurement error of the present device were analyzed using nine sample solutions, six of which were standard solutions and three of which were diluted solutions (described later). The response curves were recorded for all nine solutions. The response times of the present system and glass electrode were compared using a diluted solution (6.86/100; described later). The ΔΔV measurement in the present system was performed for 50 s after the solution was switched (Figure 3). In the glass electrode, the pH values were read for 10 min and plotted at 1 min intervals. The measured values were compared with the values provided by the manufacturer of the standard solutions and the values measured by Auto-pH [14] for the diluted solutions. The values measured by Auto-pH agreed well with the values measured by a glass electrode, as previously reported [14]. 

The measurement by the glass electrode was performed using a pH meter (pH/Ion Meter F-53, Horiba, Japan) and glass electrode (Type 9611, Horiba, Japan) under a stirring condition inside a bottle. The electrode was washed with distilled water and wiped clean just before immersion into the sample solution.

### 2.6. Preparation of the Solutions 

The pH values of the standard solutions were 1.68, 4.01, 6.86, 7.41, 9.18, and 10.01. To prepare the diluted solutions, the standard solutions with pH values of 4.01, 6.86, and 9.18 were diluted with 100-fold volumes of distilled water (pH 4.01/100, 6.86/100, and 9.18/100) [13,14]. A BLS containing 60.86 μM KH_2_PO_4_, 17.39 μM Na_2_HPO_4_, and 200 mM KCl was prepared by diluting the pH 7.41 standard solution with a 500-fold volume of distilled water and then adding KCl solution. Air was bubbled into the three diluted solutions and BLS in sufficient quantity to prevent the CO_2_ in the atmosphere from changing the pH values of the solutions. The buffering capacities of the solutions have been reported in our previous papers [13,14]. All of the reagents were obtained from Wako Pure Chemical Industries, Ltd. (Osaka, Japan) and all of the measurements were performed at room temperature (23–25 °C).

## 3. Results 

### 3.1. Calibration and the Evaluation Of Linearity 

**Figure 6:**
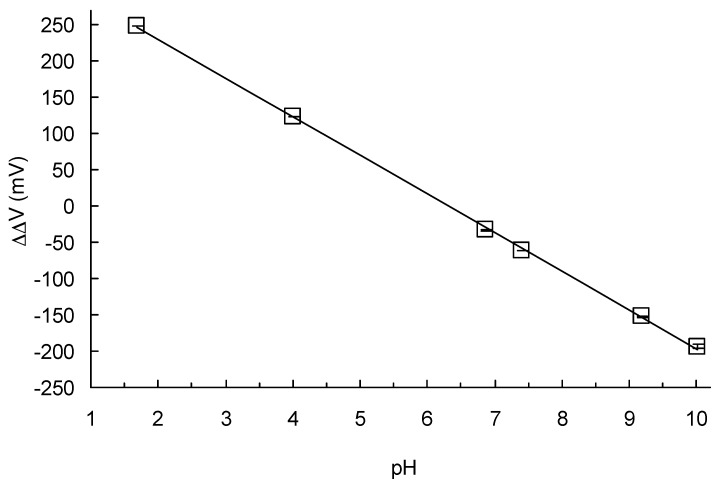
Calibration function of a microfluidic pH measurement device. Average error ± S.D. The S.D. values were 0.18, 0.58, 0.61, 0.15, 0.83, and 2.04 (mV) from the left to right.

Figure 6 shows a calibration function for the present device. The calibration line and calibration Equation (1) yielded values of pH_0_ = 6.316 and k = 53.5. The correlation factor between pH and ΔΔV was 0.9997. The maximum standard deviation (S.D.) value of the source potential, 2.04 mV, was recorded for the 10.01 standard solution. 

The k value, 53.5 mV/pH, was approximately equal to the pH sensitivity of the pH-FET used in our experiments (58 mV/pH). We are still unable to explain the small disagreements in these values. The correlation factor, 0.9997, demonstrates the high linearity of the measurements with the device in a range of pH 1.68 to 10.0. 

### 3.2. Response Time 

**Figure 7:**
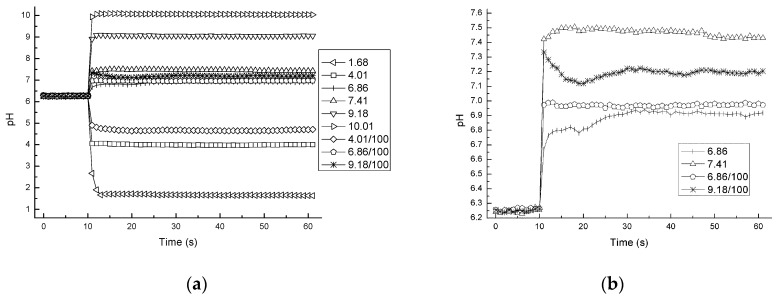
(**a**) Response curves of nine sample solutions measured by the present system; (**b**) Expansion of the *y*-axis of four sample solutions of (**a**). The data are plotted from 10 s before the solutions were switched.

Figure 7 shows the response curve of the present device using pH-FETs. The raw source potentials were converted to pH values based on the results obtained from the calibration. The 90% response times were about 2 s in solutions with high buffering capacities and solutions with low buffering capacities. As shown in Figure 7b, the pH-FET had an approximate overshoot of 0.12 pH (9.18/100) immediately after solutions with low buffering capacities were switched. The longest settling time (±1%) was 11 s (9.18/100). For reasons not known, the response of the 6.86 solution was slightly delayed compared to those of the other solutions. 

**Figure 8:**
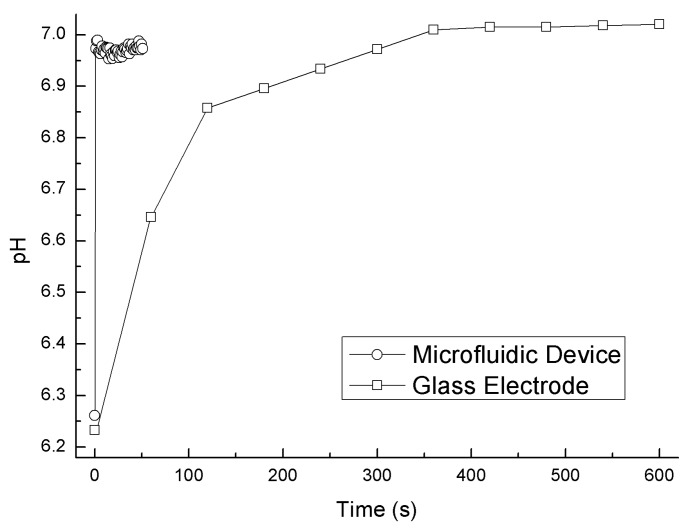
Response curves of the diluted solution (6.86/100) measured by the present system and a conventional glass electrode. The data at Time = 0 for the microfluidic device is plotted immediately before the solution for the microfluidic device was switched. The data at Time = 0 for the glass electrode is plotted immediately after the electrode was immersed in the solution for the electrode.

Figure 8 compares the response curves measured by the present system and a conventional glass electrode using the diluted solution (6.86/100). The pH values taken from the present system were plotted at intervals of 1 s. In contrast to the sensor signal of the glass electrode, which plateaued in 7 min, the signal of the present system plateaued within 20 s. 

### 3.3. pH Measurement and Evaluation of Measurement Accuracy 

**Figure 9:**
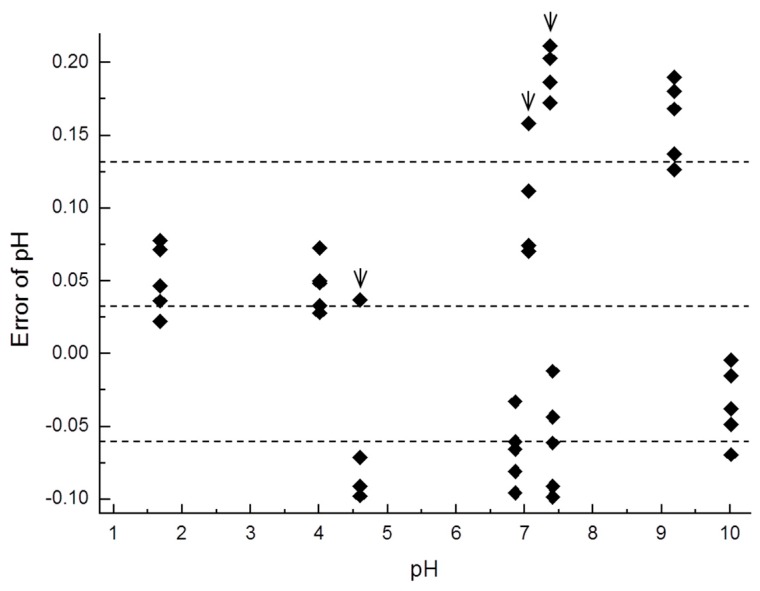
The error distribution of six standard solutions and three diluted solutions (with arrow) measured by the present system. The measurement error was 0.0343 ± 0.0974 pH (average error ± S.D., n = 42). The dotted lines show the average error and S.D.

Table 1 shows the measurement error for principal parameters in the results for the six undiluted solutions (n = 5) and three diluted solutions (n = 4). Figure 9 shows the error distribution in the measurements. The margin of error was 0.0343 ± 0.0974 pH (average error ± S.D., n = 42). The measurement error for the six undiluted solutions was 0.0158 ± 0.0849 pH (n = 30) and that for the three diluted solutions with low buffering capacities was 0.0806 ± 0.115 pH (n = 12). The measurement error and S.D. were larger for the diluted solutions than for the undiluted solutions. The measurement error was especially large for the 9.18 and 9.18/100 solutions. 

## 4. Discussion 

### 4.1. Response Time and Mechanism of Reduction 

Figure 7 demonstrates the quick response of the present device even in low-buffering-capacity solutions. Figure 8 shows the dramatically reduced response time of the present device compared to that of the glass electrode. We conjecture that the rapid response was accomplished not only by the fast response of the pH-FET sensor itself, but also the positive effect of the flowing junction [4,8,10]. The pH-FET used in this work is a catheter-tip type [21] with a gate region covered with a pH-sensitive layer of Ta_2_O_5_ and a material enabling a response time of less than 100 ms [22]. The rapid response of the present system with the flowing junction is assumed to be achieved by the following mechanism: (i) When the sample solution and BLS—two solutions with different compositions—meet, a liquid junction potential (LJP) forms due to the difference between the transport number of the electrolytes for each solution. The LJP causes measurement error, and the degree of the LJP varies in each sample solution. (ii) In a usual case, the LJP requires time to stabilize because the speed with which it forms depends on the speed with which the molecules diffuse. (iii) In the present device, the pH-FET sensor and Ag/AgCl pseudo reference electrode set near the junction picks up the sensor signal before the LJP forms. The flow of the two solutions breaks the LJP as soon as it forms, thereby maintaining a stable LJ condition during the measurement. A previous paper describes a similar mechanism in another LJ device bed as a “fresh junction” [8]. 

The overshoot of the present device was clearly lower than that of a flow-through-type sensor probe studied earlier, which overshot the readings by 0.2 pH when similar sample solutions were fed in [14]. We presume that the flowing state of the LJ is critical for this reduction in the overshoot during measurement. The area of contact between the two solutions in the channel may also be a decisive factor. This area of contact was remarkably larger in the present device than in the flow-through-type sensor probe just mentioned (about 10 mm^2^ vs. 0.23 mm^2^) [14]. For reasons we have yet to determine, the sensor signals in some of the sample solutions became unstable for periods of about 20 s after the solutions were switched.

The potential to shorten the measurement times of the present device looks promising. Judging from the stability of the response curves 20 s after the solutions were switched (Figure 7), we surmise that it may be possible to shorten the measuring period by another 20 s per sample even when leaving a margin of 10 s. Our results also indicate that the present device using pH-FET sensors responds more rapidly than the conventional method with glass electrodes even in solutions with low buffering capacities (β < 0.5 mM/pH) [13,14]. As previously mentioned, more than 7 min is required to reach a plateau during measurement of such solutions by the glass electrode method [13,14].

### 4.2. Measurement Accuracy 

We know that the flowing junction worked to reduce the measurement error, because no remarkable overshoots or delays of the response curve were observed when the solution fed to the pH-FET_m_ was switched from the BLS to a low-buffering-capacity sample solution (pH 4.01/100, 6.86/100, or 9.18/100).

As shown in Figure 9, the measurement errors were large for the 9.18 and 9.18/100 solutions. These solutions are composed of tetraborate, and the mechanism explaining the large measurement error is still unexplained. As calculated in Table 1, the measurement error and S.D. were both larger for the diluted solutions than for the undiluted solutions. This difference in measurement accuracy between the two types of solutions could stem from the pH changes caused by CO_2_, as solutions with low buffering capacities easily absorb CO_2_ in the atmosphere during measurement.

The measurement error was large for the solutions with low buffering capacities and was slightly larger overall compared to the data obtained by the flow-through-type sensor probe [13,14]. 

### 4.3. Advantages of the Present Device 

While the present system consumes BLS, the concentration of the BLS is not altered during the measurement. The concentration of the internal filling solution inside the glass electrode is increased by evaporation. This advantage presumably contributed to the reduced measurement error. 

The linearity of the sensor signal of the present system remained in the range of 1.68–10.0, indicating that calibration within a narrow range becomes unnecessary even for precise measurements. With a glass electrode, two-point calibration within a narrow range is indispensable for precise measurements due to the insufficient linearity of the signal. 

The present device has advantages compared to the glass electrodes. Thanks to its small dimensions, the pH-FET sensor used in this work can be easily packed into an outer casing made of plastic to effectively insulate the sensor body against shock. The pH-FET sensor is also made by the same mass production process used for semiconductors, assuring a reasonable manufacturing cost should the present system come onto the market in the future. While the total size of the present system may be larger than a conventional glass electrode apparatus at this trial stage, the system will be downsized by the integration of peripheral constitution elements such as a reservoir, amplifier, and microprocessor. 

### 4.4. Considerable Application of the Present Device

The pH values of environmental water are evaluated to protect the environment from adverse effects. Environmental water—such as river water, pond water, tap water, or water discharged from an industrial plant (e.g., boiler water)—often has a low buffering capacity, a condition that considerably extends the time required for precise pH measurements using a glass electrode. The superior response of the present system to solutions with low buffering capacities probably helps to shorten the measurement time and improve measurement accuracy. 

**Figure 10:**
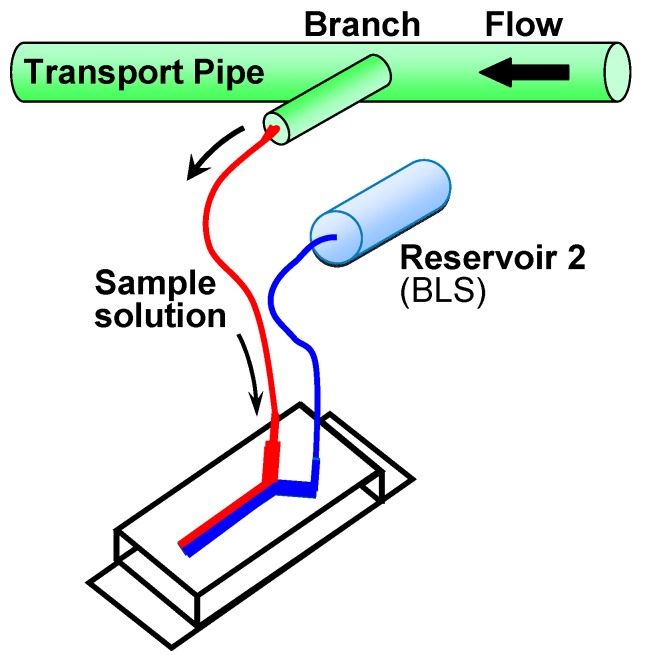
A schematic representation of application of the present system as an in-line pH measurement apparatus.

The present system could be potentially applied, for example, as an in-line pH measurement apparatus for small-scale manufacturing plants (Figure 10). Solutions in such a plant could be measured using small amounts of sample solution without any risk of contamination, as sample solution in a main pipe could be branched to the inlet by a tube, requiring no insertion of an electrode directly into the solution. 

### 4.5. Prospects for Improving the Device 

The concentration of the KCl solution needs to be more fully considered. For ease of handling, we prepared the BLS from a 200 mM KCl solution rather than a saturated (3.3 M) KCl solution, as the latter would have reduced the LJP remarkably [23]. The LJP occurring between the sample solution and KCl solution is known to cause measurement errors due to the variation in the LJP resulting from the changing compositions of the sample solution [4,5,6,7,8]. In this research, we obtained superior measurement accuracy, presumably because the flows of the sample solution and BLS decreased the LJP between the solutions. The LJP could probably be further reduced by increasing the concentration of KCl in the BLS.

Many factors can improve the measurement performance of the present device. The dimension of the channel, for example, is presumed to affect the flow characteristics. The surface characteristics of the channel, such as the roughness and surface energy, are also critical to the fluid flows. The response time, measurement stability, and measurement accuracy may be improved by optimizing these parameters. Direct measurement of the LJP is essential, but the very narrow width of the channel hinders the measurement reliability. While pH-FET_r_ is not crucial for measurement, the present device composed of a differential measurement circuit with two pH-FETs and an Ag/AgCl exhibits superior performance against noise and changes of temperature.

We expect that rearrangements of the pH-FETs and channel will allow further miniaturization of the LJ device. The integration of reservoirs into the main body of the measurement device also seems likely. The required sample volume can be reduced by narrowing the channel further.

## 5. Conclusions

We have combined a microfluidic device with pH-FETs to construct a new pH measurement system with a flowing-type LJ device. The present device had a shorter response time than the glass electrode in the pH measurement for the low-buffering-capacity solution. The method presented in this paper will be useful for the measurement of pH in fields of environmental science. 

## Figures and Tables

**Table 1:** pH measurement error using the present system.

Number	Solution (True Value)	ΔΔV (mV)	pH	Error of pH	Calibration *
1	1.68	236.03	1.644	0.036	1
2	(1.68)	233.22	1.658	0.022	2
3		234.44	1.634	0.046	2
4		236.03	1.602	0.078	2
5		235.72	1.608	0.072	2
6	4.01	117.86	3.960	0.050	1
7	(4.01)	116.27	3.960	0.050	2
8		115.24	3.982	0.028	2
9		115.48	3.977	0.033	2
10		117.49	3.937	0.073	2
11	6.86	−33.45	6.925	−0.065	1
12	(6.86)	−33.94	6.921	−0.061	2
13		−35.71	6.955	−0.095	2
14		−32.53	6.893	−0.033	2
15		−34.97	6.941	−0.081	2
16	7.41	−62.81	7.501	−0.091	1
17	(7.41)	−61.89	7.471	−0.061	2
18		−63.78	7.509	−0.099	2
19		−59.39	7.422	−0.012	2
20		−60.98	7.453	−0.043	2
21	9.18	−139.89	9.012	0.168	1
22	(9.18)	−138.98	8.990	0.190	2
23		−139.47	9.000	0.180	2
24		−142.21	9.054	0.126	2
25		−141.66	9.043	0.137	2
26	10.01	−193.30	10.06	−0.049	1
27	(10.01)	−190.98	10.01	−0.004	2
28		−194.28	10.08	−0.069	2
29		−192.69	10.05	−0.038	2
30		−191.53	10.03	−0.015	2
31	4.01/100	75.99	4.696	−0.097	3
32	(4.599)	77.27	4.670	−0.071	3
33		82.52	4.562	0.037	3
34		76.30	4.690	−0.091	3
35	6.86/100	−35.10	6.984	0.075	3
36	(7.059)	−31.01	6.900	0.159	3
37		−33.26	6.947	0.112	3
38		−35.28	6.988	0.071	3
39	9.18/100	−44.92	7.187	0.186	3
40	(7.373)	−45.59	7.201	0.172	3
41		−43.70	7.162	0.211	3
42		−44.13	7.171	0.202	3
Averaged Error of pH ± S.D.					
	1–42 (all)		0.0343 ± 0.0974		
	1–30 (Non-diluted)		0.0158 ± 0.0849		
	31–42 (Diluted)		0.0806 ± 0.115		

* (1/k, pH_0_) = 1: (−0.0196, 6.270); 2: (−0.0197, 6.252); 3: (−0.0206, 6.262).
